# Introduction of Ultrasound Simulation in Medical Education: Exploratory Study

**DOI:** 10.2196/13568

**Published:** 2019-09-26

**Authors:** Selim Hani, Gihad Chalouhi, Zavi Lakissian, Rana Sharara-Chami

**Affiliations:** 1 Department of Industrial Engineering American University of Beirut Beirut Lebanon; 2 International Society of Ultrasound in Obstetrics and Gynecology London United Kingdom; 3 SimECHOLE Paris France; 4 Division of Maternal Fetal Medicine Department of Obstetrics and Gynecology American University of Beirut Medical Center Beirut Lebanon; 5 Simulation Program Faculty of Medicine American University of Beirut Medical Center Beirut Lebanon; 6 Department of Pediatrics and Adolescent Medicine American University of Beirut Medical Center Beirut Lebanon

**Keywords:** medical education, simulation training, ultrasonography

## Abstract

**Background:**

Ultrasound is ubiquitous across all disciplines of medicine; it is one of the most commonly used noninvasive, painless diagnostic tools. However, not many are educated and trained well enough in its use. Ultrasound requires not only theoretical knowledge but also extensive practical experience. The simulated setting offers the safest environment for health care professionals to learn and practice using ultrasound.

**Objective:**

This study aimed to (1) assess health care professionals’ need for and enthusiasm toward practicing using ultrasound via simulation and (2) gauge their perception and acceptance of simulation as an integral element of ultrasound education in medical curricula.

**Methods:**

A day-long intervention was organized at the American University of Beirut Medical Center (AUBMC) to provide a free-of-charge interactive ultrasound simulation workshop—using CAE Vimedix high-fidelity simulator—for health care providers, including physicians, nurses, ultrasound technicians, residents, and medical students. Following the intervention, attendees completed an evaluation, which included 4 demographic questions and 16 close-ended questions based on a Likert scale agree-neutral-disagree. The results presented are based on this evaluation form.

**Results:**

A total of 41 participants attended the workshop (46% [19/41] physicians, 30% [12/41] residents, 19% [8/41] sonographers, and 5% [2/41] medical students), mostly from AUBMC (88%, 36/41), with an average experience of 2.27 (SD 3.45) years and 30 (SD 46) scans per attendee. Moreover, 15 out of 41 (36%) participants were from obstetrics and gynecology, 11 (27%) from internal medicine, 4 (10%) from pediatrics, 4 (10%) from emergency medicine, 2 (5%) from surgery and family medicine, and 5 (12%) were technicians. The majority of participants agreed that ultrasound provided a realistic setting (98%, 40/41) and that it allowed for training and identification of pathologies (88%, 36/41). Furthermore, 100% (41/41) of the participants agreed that it should be part of the curriculum either in medical school or residency, and most of the participants approved it for training (98%, 40/41) and teaching (98%, 40/41).

**Conclusions:**

All attendees were satisfied with the intervention. There was a positive perception toward the use of simulation for training and teaching medical students and residents in using ultrasound, and there was a definite need and enthusiasm for its integration into curricula. Simulation offers an avenue not only for teaching but also for practicing the ultrasound technology by both medical students and health care providers.

## Introduction

### Background

Ultrasound is ubiquitous across all disciplines of medicine; it is one of the most commonly used noninvasive, painless diagnostic tools. However, not many are educated and trained well enough in its use. In obstetrics and gynecology (OBGYN), for instance, ultrasound is the primary method of imaging [1]. Its use encompasses screening as well as expert examination of normal and abnormal cases [2]. It has become an essential part of medical practice, often irrespective of the ability, competence, and experience of the operators [3,4]. The lack of standardization in training and assessment of skills has become a matter of concern worldwide [5].

Currently, theoretical knowledge of ultrasound technology and application is sometimes insufficient, and practical training has traditionally been patient-dependent, that is, achieved on actual patients or volunteers [6]. However, this conventional approach has numerous challenges, especially during the initial phase of training; it adds undue pressure on trainees interacting with patients, potentially distracting them from correctly handling the ultrasound probe and/or accurately interpreting the images [6]. Furthermore, developing competency in ultrasound is largely dependent on the variety and number of cases encountered during clinical practice [7]. Finally, the more important issue is the challenge of patients not willing to be examined by trainees [8]. Ultrasound training is time-consuming and requires extensive teaching resources [3,4]. Consequently, some trainees may never acquire the basic skills and knowledge needed for independent practice [5]. The lack of sufficient operator skills can lead to diagnostic errors that may compromise patient safety. The increased focus on medical errors and patient safety calls for development of alternative methods for continuous education and assessment of skills [9].

These changes in the context of medical education and training have paved the way for a somewhat new concept of learning, that is, simulation, focused mainly on learners’ needs and patient safety [10]. The emerging field of simulation-based education has been shown to improve basic ultrasound training [2-5]. Simulation provides a safe, controlled, and learner-centered environment, which allows for repeated practice without any patient discomfort or harm [6,7]. Simulation-based training may enable trainees to become familiar with image optimization, probe orientation, as well as practicing a systematic approach to ultrasonography before beginning clinical training [5-8].

Ultrasound simulators are integrated simulators, generally composed of a human mannequin, a mock probe, and a computer. Usually, the mock probe is connected directly to a monitor that displays the ultrasound image depending upon the probe’s position and movements. Most of these simulators use electromagnetic tracking systems to define the probe’s position. The mock probe usually contains a 3-dimensional sensor, capable of acquiring virtual position data instantaneously [7,11-15]. These simulators have been applied mainly in teaching the basic skills of cardiac ultrasound examination to students and residents in emergency medicine and in internal medicine. Over the last few years, several studies have investigated the effectiveness of simulation-based echocardiography training compared with conventional methods such as theoretical lectures and hands-on training on patients. Findings of these studies suggest that the use of echocardiographic simulators gave very positive results regarding motivation and a decrease in anxiety compared with examination of real patients [16]. The use of transesophageal echocardiographic simulation proved not only to be realistic and helpful [17] but also to be superior to conventional methods of teaching [16-18]. Simulation has also been found to be helpful for introducing surgery residents to the use of ultrasound in trauma cases [19]. It has been established that there is improvement in knowledge and better recognition of clinical scenarios after training sessions on the simulator [20]. However, a study by Cawthorn et al [21] underlines the importance of supervised training using simulation, stating the necessity of combining both teaching methods.

### Objectives

**Figure 1:**
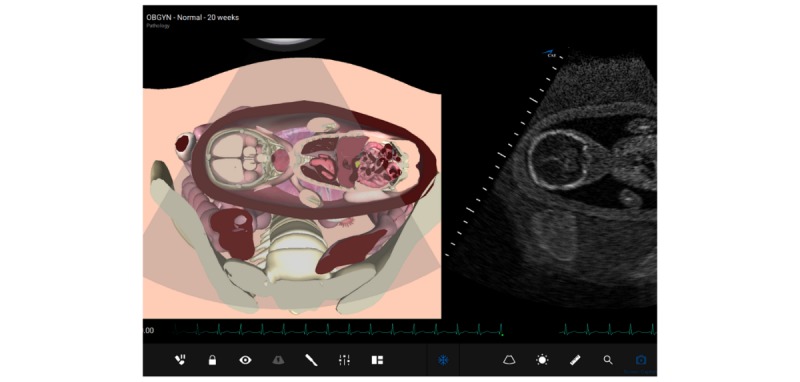
CAE Vimedix high-fidelity simulator.

To justify the expenses of adding a costly, albeit proficient and high-fidelity simulator, the authors needed to assess stakeholders’ interest and institutional need for the investment. Therefore, a day-long workshop was organized to provide a free-of-charge interactive ultrasound simulation training—using CAE Vimedix high-fidelity simulator (see Figure 1)—for health care providers, including physicians, nurses, ultrasound technicians/sonographers, residents, and medical students. Our aim for this intervention was to assess the readiness and need of health care professionals to practice using ultrasound via simulation and to estimate their perception and acceptance of simulation as an integral element of medical education curricula, particularly in relation to teaching and practicing the use of US.

## Methods

### Design

The study is an experimental intervention, that is, an ultrasound simulation workshop provided at the American University of Beirut Medical Center (AUBMC).

### Participants

An open invitation to the event was circulated via email; participants included physicians, nurses, ultrasound technicians/sonographers, residents, and medical students.

### Procedures

CAE Vimedix high-fidelity simulator was used for the workshop. This simulator facilitates engaging and intuitive learning in cardiac, pulmonary, abdominal, and OBGYN US—all in 1 common platform. With its state-of-the-art manikin-based system and innovative software tools, CAE Vimedix accelerates the development of essential psychomotor and cognitive skills for ultrasound probe handling, image interpretation, diagnoses, and clinical decision making (CAE Healthcare, Corp, 2019).

The workshop was divided into 4 modules. All modules started with a short didactic presentation of the theoretical basis to ultrasound relating to that specific module (10 min). The first module contained adult cardiology scenarios (pulmonary stenosis, cardiac tamponade, heart failure, and aortic regurgitation). The second module contained emergency medicine topics (pneumonia, acute myocardial infarction, pleural effusion, pneumothorax, and acute abdomen). The third module contained pediatric cardiology topics (Ebstein anomaly, valvular diseases, and single ventricle physiology). The fourth module was tailored for OBGYN and emergency medicine providers, and it contained scenarios on ectopic pregnancy (8 weeks), normal fetus (8 weeks and 12 weeks), and cleft lip (20 weeks). The participants got a 1-hour hands-on practice with direct one-on-one feedback during each module.

Following the intervention, the attendees were asked to complete an evaluation, which included 4 demographic questions and 16 close-ended questions based on a Likert scale (agree-neutral-disagree).

### Measuring Impact

Novel training strategies should ideally create a chain of impact at several levels. The most widely used training evaluation methodology is the Kirkpatrick and Phillips model [22,23], which measures training outcomes at 5 levels, starting at reaction/planned action and ending with return on investment (ROI):

1. *Level 1—Reaction and satisfaction*: this measures participants’ reaction to and satisfaction with the training, usually measured in surveys, and their planned action (their plans to use what they have learned).
2. *Level 2—Learning*: this assesses how much participants have learned (with pre- and posttests).
3. *Level 3—Behavior, application, and implementation*: this assesses whether the skills and knowledge gained in training are applied and practiced in the workplace or have changed learners’ behavior.
4. *Level 4—Results*: this measures the extent to which the institutions’ measures (output, quality, costs, and time) have improved after training; although this can be considered as the goal of a strategy, it is important to go beyond this level of evaluation to verify that the program’s costs do not outweigh its benefits.
5. *Level 5—Return on investment*: this compares the benefits from the program with its cost [24,25] and is the ultimate level of evaluation.

The evaluation of ultrasound simulation has until now remained mainly at levels 1 and 2. Most studies have evaluated reaction, satisfaction [25], or learning [17,22]. Currently, several ultrasound simulators measure time to complete tasks and accuracy of procedure; however, most studies have not yet evaluated the transfer of knowledge acquired during simulation training into clinical practice [26]. In addition to these measurable benefits, most training programs have intangible benefits, including stress reduction and increased commitment of trainees, improved patient satisfaction, less patient complaints, as well as decline or avoidance of conflict [25]. Our study primarily targeted level 1.

### Analysis

Data collected from the evaluations were entered, coded, and analyzed via the Statistical Package for Social Sciences version 24 (IBM Corp). Descriptive analyses were performed using the number and percentage for categorical variables or mean and SD for continuous ones. To avoid redundancy, the 5-point Likert scale was collapsed into 3 points: strongly agree and agree were combined under “agree,” and similarly, strongly disagree and disagree were combined under “disagree;” therefore, analyses were performed on the scale agree-neither agree nor disagree-disagree.

## Results

### Participant Demographics

A total of 41 participants attended the workshop (46% [19/41] physicians, 30% [12/41] residents, 19% [8/41] sonographers, and 5% [2/41] medical students), mostly from AUBMC (88%, 36/41), with an average experience of 2.27 (SD 3.45) years and 30 (SD 46) scans per attendee. Moreover, 36% (15/41) of participants were from OBGYN, 27% (11/41) from internal medicine, 10% (4/41) from pediatrics, 10% (4/41) from emergency medicine, 5% (2/41) from surgery and family medicine, and 12% (5/41) were technicians.

### Participant Response to Ultrasound Simulation Training

Overall, Twenty participants had been previously exposed to simulation in general. The majority of participants agreed that ultrasound simulation provided a realistic setting (98%, 40/41) and that it allowed for training and identification of pathologies (88%, 36/41). In addition, 100% (41/41) of the participants agreed that it should be part of the curriculum either in medical school or residency, and most of the participants agreed that it was useful for training (98%, 40/41) and teaching (98%, 40/41; Table 1).

**Table 1:** Results of the evaluation forms (N=41).

Evaluation questions	Agree, n (%)	Neither, n (%)	Disagree, n (%)
In terms of complexity...pathologies on the simulator seemed significantly less complex	39 (95)	1 (2)	1 (2)
Simulation-based assessment of US^a^ skills is an acceptable method for evaluation	39 (95)	1 (2)	1 (2)
The US simulation gives realistic images, and the pathologies are represented realistically	38 (93)	2 (5)	2 (5)
The US simulation gives a realistic sensation of probe manipulation	38 (93)	2 (5)	1 (2)
The US simulation should be introduced as part of the US training in the medical school curriculum	41 (100)	—^b^	—
The US simulation is a good tool for training	40 (98)	1 (2)	—
The US simulation is a good tool for teaching	40 (98)	1 (2)	—
The US simulation allows training and identification of complex or /rare pathologies	36 (88)	3 (7)	2 (5)
On the basis of this session, I do not see any added value of the US simulation, and there is no justification for its use in medical school environments	9 (22)	1 (3)	31 (75)
The US simulation allows for good auto-evaluation of health care professionals	39 (95)	2 (5)	—
Handling of the US session on the simulation requires the same level of care and meticulousness as the process with a real patient	25 (62)	8 (20)	7 (17)
Handling a case on the US simulation is as stressful as real-life patients	11 (27)	4 (10)	26 (63)
Simulation-Based Assessments should be used for future licensing exams	35 (85)	6 (15)	—
An US simulation allows exposure of students/professionals to a wider range of pathologies	39 (95)	1 (2)	1 (2)
This session was satisfactory	39 (95)	1 (2)	1 (2)
Participation in future simulation initiatives	40 (98)	1 (2)	—

^a^US: ultrasound.

^b^Cells with 0 responses. For example, when 100% of participants responded with “agree” and none with “neither” or “disagree.”

## Discussion

### Principal Findings and Conclusions

Our findings showed that participants unanimously supported the introduction of ultrasound via simulation in medical school curricula and residency programs. The importance of hands-on repeat-training and deliberate practice [27] until proficiency is reached has superseded and surpassed the outdated paradigm of “see one, do one, teach one” [28]. So far, there is no consensus or standardization of the teaching or training of ultrasound among different institutions and countries for educational purposes or for assessment of practitioners’ skills and accreditation [5]. Given the high variability between learners in the time and training needed to gain proficiency, it is unlikely that a minimum set number of scans can adequately reflect candidates’ skills; some trainees reach a level of competency that is suitable for clinical practice after a few scans, whereas others need more time to reach the same level [29,30]. Our intervention showed that simulation-based ultrasound training could provide a relatively realistic setting for training, assessment, and practice. However, further research is needed to assess the retention of knowledge and skills by the workshop participants.

There is broad consensus on the utility of integrating virtual reality into ultrasound education and into training programs [5]. It has been proposed as a valid and reliable method for assessment of skills [29,30]. Simulation, however, is not meant to replace clinical training and tutoring [26]; instead, it offers a complementary useful method for introducing trainees to ultrasound practice, allowing them to become familiar with image optimization and probe orientation, without being confronted with the stresses of the clinical setting.

There are a number of commercially available ultrasound simulators, but they remain expensive and require maintenance and adequate training for their use. These factors may limit the widespread adoption of the technology. Some practitioners believe that acquisition of simulators can be economically beneficial by allowing trainees to improve their performance without monopolizing ultrasound machines required in the clinical setting [5]. However, proper cost-effectiveness analyses have to be conducted to verify and substantiate these claims.

### Limitations

We acknowledge that the study has limitations, including the fact that it is an analysis of 1 workshop. The heterogeneity among participants in terms of disciplines, experience, and specialty lead us to consider our findings relatively sound in external validity. More importantly, future interventions and assessments need to be conducted to measure the long-term effects of such exercises on participants’ knowledge and skill retention.

## Abbreviations

AUBMC: American University of Beirut Medical Center
OBGYN: obstetrics and gynecology
ROI: return on investment
US: ultrasound
